# Efficacy and Prognosis of ROSA Robot-Assisted Stereotactic Intracranial Hematoma Removal in Patients with Cerebral Hemorrhage in Basal Ganglia Region: Comparison with Craniotomy and Neuroendoscopy

**DOI:** 10.1007/s12975-025-01330-8

**Published:** 2025-02-01

**Authors:** Haitao Wu, Bin Lu, Wei Wang, Xiaoyi Wang, Tingxuan Wang, Yue Bao, Luo Li

**Affiliations:** 1 Qingdao University Medical College, Qingdao University, Qingdao, Shandong China; 2 Department of Neurosurgery, Qingdao Hospital, University of Health and Rehabilitation Sciences (Qingdao Municipal Hospital), No.5 Donghai Zhong Road, Qingdao, 266000 Shandong China

**Keywords:** Robot-assisted stereotactic assistance, Neuroendoscopy, Craniotomy, Intracranial hematoma, Basal ganglia, Clinical efficacy, Prognosis

## Abstract

This study compares the clinical efficacy and outcomes of three surgical techniques—robot-assisted stereotactic assistance (ROSA), neuroendoscopy, and craniotomy—in the removal of intracranial hematomas in patients with cerebral hemorrhage affecting the basal ganglia. This retrospective study included 110 patients, who were grouped based on the surgical method used: 40 patients in the ROSA group, 50 in the craniotomy group, and 20 in the endoscopy group. We then compared the outcomes of the ROSA group with those of the craniotomy and endoscopy groups. Compared with the craniotomy group, the ROSA group had a significantly shorter operation time, higher hematoma clearance rate, lesser intraoperative blood loss, fewer postoperative pulmonary infections, and lower modified Rankin Scale (mRS) score at discharge and > 3 months after discharge. Compared with the endoscopy group, the ROSA group had a shorter operation time, lesser intraoperative blood loss, and fewer intraoperative blood transfusions. The ROSA robot provided superior surgical outcomes and patient prognoses compared to craniotomy and neuroendoscopy for the removal of intracranial hematomas in patients with basal ganglia cerebral hemorrhage.

**Supplementary Information:**

The online version contains supplementary material available at 10.1007/s12975-025-01330-8.

## Background

Cerebral hemorrhage has high morbidity and mortality rates [1]. Nearly one-third of patients die within 1 month [2] and only approximately 20% achieve full recovery within 6 months after clinical treatment [3]. The presence of a hematoma following a cerebral hemorrhage increases intracranial pressure, which can result in cerebral herniation and altered blood flow. Additionally, the surrounding edema continues to exert pressure on the brain, exacerbating damage over time [4]. Therefore, surgical hematoma removal to reduce intracranial pressure is an effective treatment modality [5]. However, craniotomy may not be the best method for hematoma removal in cases with cerebral hemorrhage [3, 4]. Although craniotomy helps reduce intracranial pressure, it carries risks such as damage to healthy brain tissue during the procedure, significant intraoperative blood loss, and potential side effects from anesthesia drugs [3]. The current surgical treatments for cerebral hemorrhage mainly include craniotomy to remove hematoma, neuroendoscopic removal, and minimally invasive drainage of the hematoma [6]. Minimally invasive surgery has the obvious advantages of reduced surgical trauma, higher positioning accuracy, and shorter operation time compared with traditional craniotomy [3]. Rebleeding and intracranial infection rates after robot-assisted cerebral hemorrhage are also significantly lower than those after open hematoma removal, and patients have a better prognosis [7]. The robotic surgical assistant (ROSA) stereotactic robot features a frameless surgical robot that integrates neuronavigation, stereotactic localization, and systematic operation; it can also use instruments for high-precision localization, three-dimensional hematomas reconstruction, and manipulation of the robotic arm for minimally invasive drilling and drainage of intracerebral hematomas.

We analyzed clinical data to compare the efficacy and outcomes of ROSA, craniotomy, and neuroendoscopic techniques for hematoma removal in patients with basal ganglia intracerebral hemorrhage.

## Patients

The clinical data of patients who underwent surgical treatment for cerebral hemorrhage in the basal ganglia at the Department of Neurosurgery of Qingdao Municipal Hospital between March 2020 and March 2024 were retrospectively analyzed. The inclusion criteria were (1) age 18–80 years; (2) Glasgow Coma Score (GCS) ≥ 5 points; (3) preoperative cranial computed tomography (CT) findings suggesting cerebral hemorrhage in the basal ganglia region, with a hematoma volume > 20 mL; and (4) no coagulation dysfunction. The exclusion criteria were as follows: (1) a combination of contagious meningitis, pulmonary infection, and coagulation function abnormalities; (2) GCS on admission < 5 points; (3) intracranial hemorrhage due to intracranial aneurysm, arteriovenous malformation, craniocerebral trauma, or tumor stroke; (4) brainstem hemorrhage; (5) recent use of aspirin and other medications; and (6) preoperative cerebral herniation, massive intraventricular hemorrhage, or obstructive hydrocephalus. The Institutional Review Board of Qingdao Municipal Hospital approved this study and all procedures were performed in accordance with the guidelines of the Declaration of Helsinki. A total of 110 patients met the inclusion criteria and were included in this study. The patients were divided into groups according to surgical technique, with the ROSA, craniotomy, and neuroendoscopy groups including 40, 50, and 20 cases, respectively.

We collected clinical data from patients, including age, gender, Glasgow Coma Scale (GCS) upon admission, and medical history of hypertension, diabetes, and coronary artery disease. Additionally, we gathered treatment-related data, such as operation time, intraoperative blood transfusion, hematoma clearance rate, and postoperative complications (e.g., pulmonary infection, urinary tract infection, intracranial infection, rebleeding, and postoperative ventilator-assisted ventilation > 96H). We also recorded postoperative mortality, surgical and hospitalization costs, the time from surgery to discharge, and the modified Rankin Scale (mRS) scores at discharge and more than three months after discharge.

## Surgical Procedures (Methods)

### ROSA

**Fig. 1:**
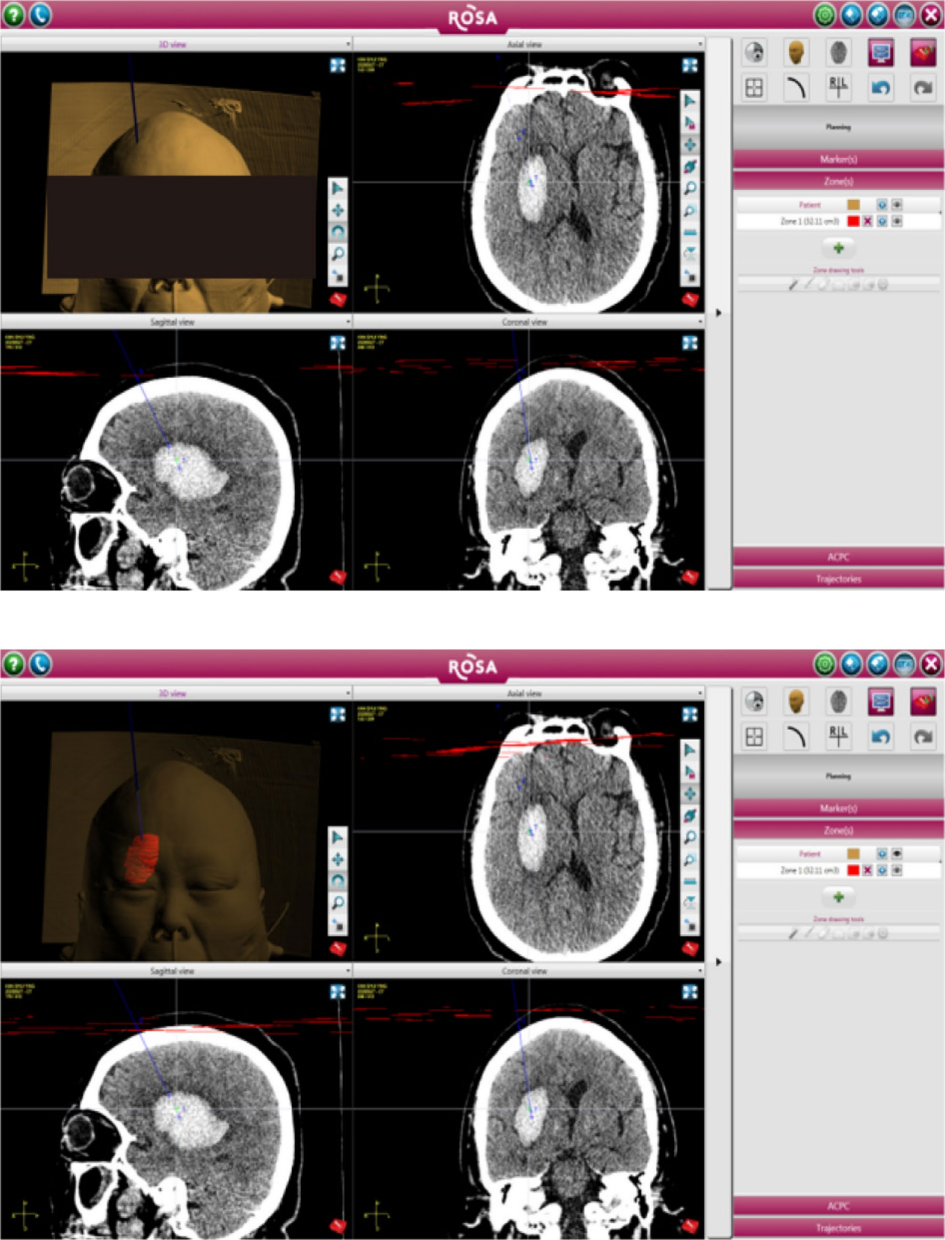
The location and 3D reconstruction of cerebral hematoma were performed with imaging images

Preoperative planning: Each patient underwent a preoperative whole-head thin-layer CT scan (layer thickness, 0.625 mm) with a sweep from the crown to the tip of the nose. The original DICOM data were imported into the ROSA surgical planning workstation and three-dimensional (3D) image reconstruction was performed (Fig. 1). The hematoma volume was calculated, and the puncture needle tract was designed according to the specific hematoma site and shape. Transfrontal puncture was selected for hemorrhages in the basal ganglia region, with a starting point located 2–4 cm in front of the coronal suture and 2.5–3 cm beside the midline. The target puncture point was slightly below the longitudinal axis of the center of the hematoma. The puncture needle channel was parallel to the long axis of the hematoma, and the needle channel design avoided the blood vessels, cerebral sulcus, and lateral ventricles.

**Fig. 2:**
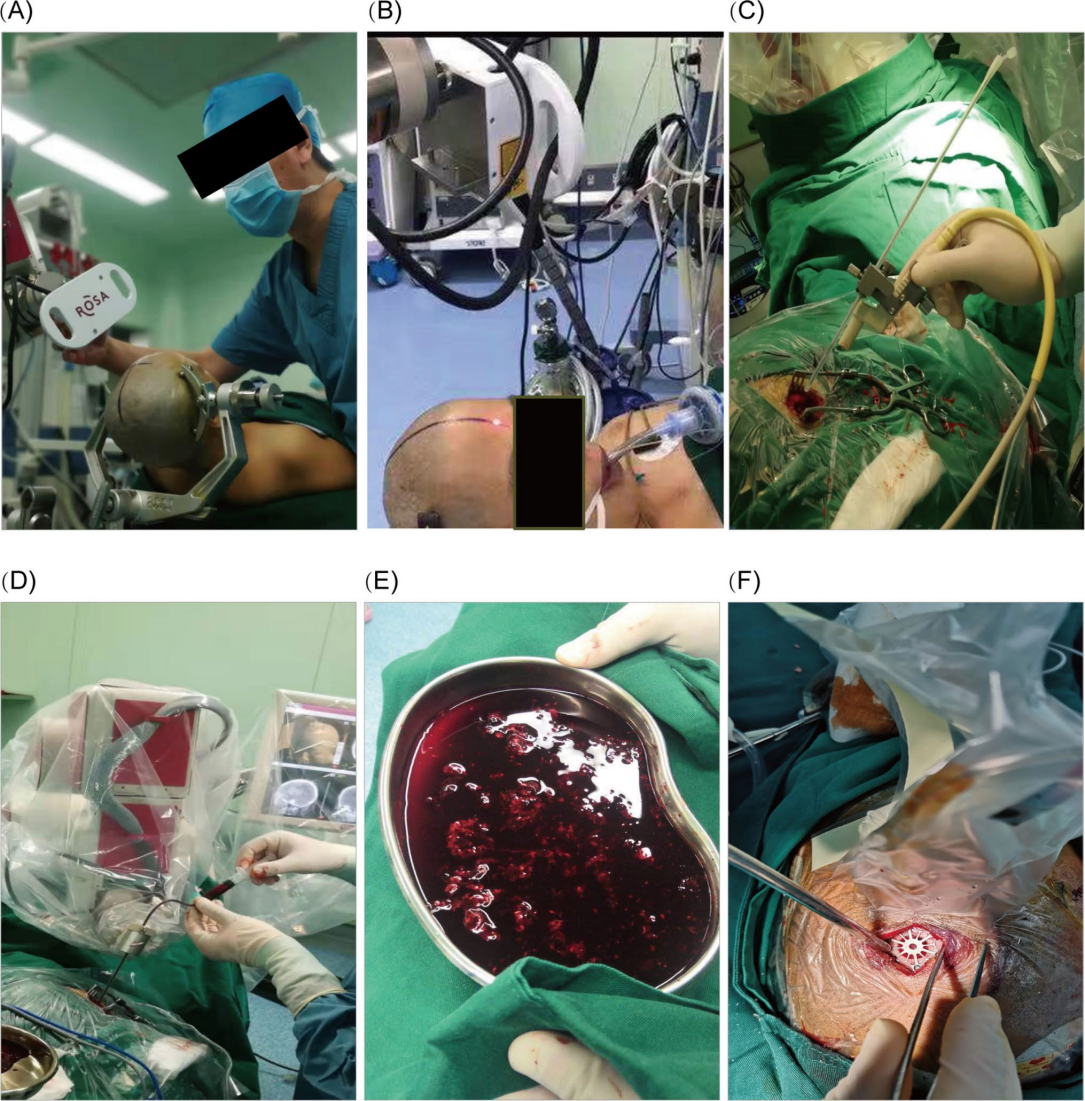
ROSA robot assisted removal of cerebral hematoma operation process

**Fig. 3:**
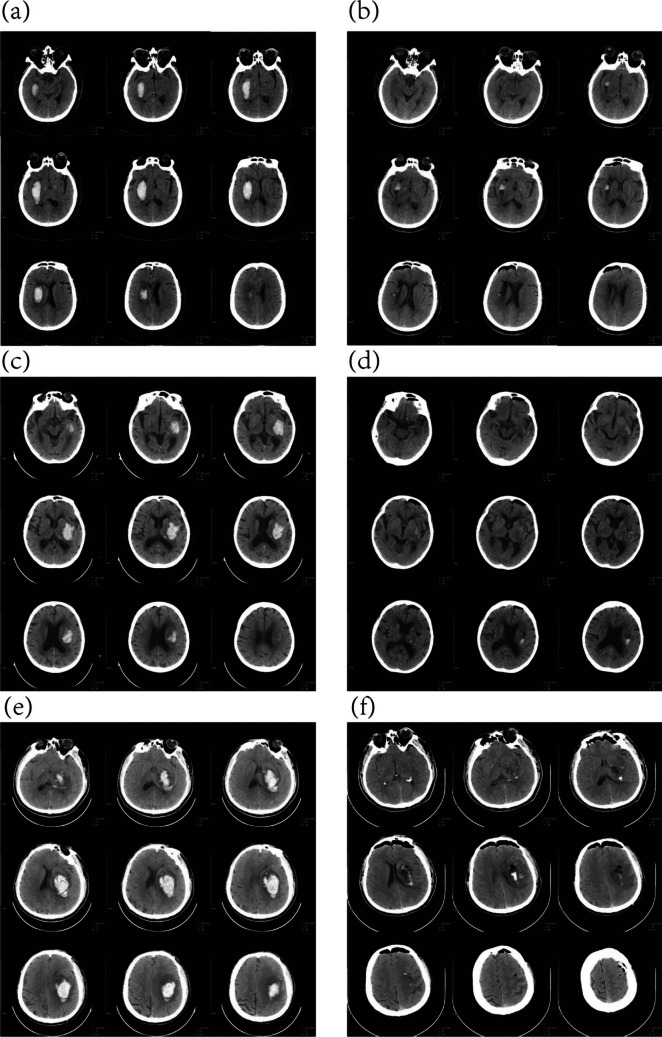
The CT images immediately after ROSA robot-assisted stereotactic removal of intracranial hematoma were compared with CT that before surgery.( First patient:a,b; Second patient:c,d; The third patient:e,f)

Intraoperative procedure: After tracheal intubation and general anesthesia, each patient was placed in the supine position on the surgical bed with the head fixed with a head frame. The ROSA robot was then fixed; facial laser registration was performed (Fig. 2A); the bony nasal tip, nasal root, inner and outer canthus, eyebrow arch, and other markers were collected; the face was scanned alone at 5,000–8,000 sites (Fig. 2B). The error was manually adjusted to complete the fusion of CT and the patient's face, with the precision of the system controlled to within 1 mm. According to the puncture site design for approximately 3 cm long scalp incision, routine disinfection was performed after placing an aseptic towel and making a surgical patch. The scalp was then incised and fixed with a retractor. A hole was then drilled into the skull and enlarged to approximately 2 cm in diameter using a milling drill. Bone wax was used to seal blood leakage from the barrier, and the dura was incised in a cross shape after bipolar electrocoagulation of the dural surface blood vessels. A No. 10 extraventricular drain with a 1.5 mm inner diameter and 2 mm outer diameter was chosen as the puncture hose, with four lateral holes at the head of the drain, and a 1.5-mm-diameter Kirschner needle inserted inside the puncture hose for support (Fig. 2C). The ROSA robotic arm was then moved to the working position according to the preoperatively planned puncture path. Under robotic arm guidance, the end of the puncture hose tip was placed at the target point of the hematoma and the guide needle was withdrawn. A 5 ml syringe was then used to manually aspirate the hematoma, with the strength of the aspiration controlled to avoid forcing aspiration when resistance was encountered (Fig. 2D, E ). The degree of hematoma removal was calculated according to the preoperative hematoma volume. Intraoperative ultrasonography was used to detect residual hematoma. The depth and angle of the puncture tube were adjusted based on the location of the residual hematoma, and warm saline was used to repeatedly flush and replace the residual hematoma. After withdrawing the puncture tube, the "snowflake" connecting piece was used to block the bone hole (Fig. 2F), the scalp was sutured layer-by-layer, and sterile auxiliary materials were used for bandaging. Immediately after surgery, cranial CT was repeated to observe any new issues in the blood and calculate the hematoma clearance rate ((Fig. 3).

## Craniotomy

Each patient was positioned supine on the operating table after general anesthesia and tracheal intubation. The head was rotated approximately 30° away from the hematoma. An arc-shaped frontotemporal incision, 12–15 cm in length, was made. The surgical area was disinfected and sterilized. The scalp and temporalis muscles were dissected layer by layer, retracted to the nasal side, and secured with a scalp retractor. After drilling, a skull flap measuring approximately 6 × 8 cm was created using a milling cutter. Bone wax was applied to seal the bone edges, and the dura mater was suspended along the bone border. The dura was then incised radially, and intraoperative ultrasonography was performed to locate the hematoma. The shortest path from the cortex to the hematoma was identified, avoiding critical functional areas. Access was made through the middle temporal gyrus or insular lobe. The hematoma was removed using suction and bipolar coagulation, and the surgical cavity was packed with hemostatic material to control bleeding. The dura was sutured with a watertight seal, the skull flap was repositioned, and the temporal muscles and scalp were sutured layer by layer.

## Neuroendoscopy

Each patient was placed in the supine position on the operating table after general anesthesia and tracheal intubation. A straight frontal incision, approximately 5–6 cm in length, was made 2–3 cm anterior to the coronal suture and 2–3 cm along the midline. Routine disinfection, sterile towels, and surgical drapes were used. The scalp, galeal aponeurosis, and periosteum were carefully dissected layer by layer and secured with retractors. A burr hole was drilled into the skull, and a circular bone window, approximately 3 cm in diameter, was created using a milling cutter. Bone wax was applied to seal the edges, and the dura was suspended around the bone window before being incised in a “cross” pattern. Intraoperative ultrasound was used to locate and assess the hematoma's depth. A fusiform tissue expansion balloon was inserted into the hematoma cavity and gradually expanded to establish the surgical channel. A transparent working sheath was then inserted deep into the hematoma along this channel, and the intrathecal puncture device was removed. A 0° or 30° neuroendoscope was placed in the working sheath, and the hematoma was carefully removed by suction in a clockwise direction, starting from deep areas and moving outward. During this process, the boundary between the hematoma and white matter was closely monitored. After checking for any active bleeding or remaining hematoma around the working sheath, warm saline was used to flush the surgical cavity. Fluid gelatin was introduced to stop any bleeding, and the working sheath was removed. The patients were monitored for signs of cerebral cortex collapse, ensuring proper pulsation and confirming that decompression was achieved. The dura was sutured with a watertight seal, the skull bone flap was repositioned and fixed, the periosteum was reset, and the scalp was sutured layer by layer.

## Hematoma Volume [8]

Hematoma volume was calculated using Broderick's method 8 (length × width × height /2).

## Hematoma Clearance Rate

(Preoperative hematoma volume—postoperative hematoma volume)/preoperative hematoma volume × 100%.

## Statistical Analysis

Statistical analysis was performed using SPSS 27.0 software and R language 4.2. Categorical variables were presented as frequencies, and differences were compared using the chi-square test or Fisher's exact test. Continuous variables were expressed as mean ± standard deviation. Normally distributed continuous variables were compared using t-tests based on Kolmogorov–Smirnov statistics, while non-normally distributed variables were analyzed for intergroup differences using the Mann–Whitney U test. All statistical tests were two-tailed, with a significance level set at *P* < 0.05.

### Baseline Characteristics of All Patients with Intracerebral Hemorrhage in the Basal Ganglia

Following the inclusion and exclusion criteria, a total of 110 patients were included in this study, 40 patients in ROSA group, 50 patients in craniotomy group and 20 patients in endoscopy group. **ROSA group**: The age of the patients was 59.80 ± 12.53 years, 26 (65.00%) were male patients and 14 (35.00%) were female patients. There were 3 patients (7.50%) with coronary artery disease, 26 patients (65.00%) with hypertension and 5 patients (12.50%) with diabetes mellitus. The GCS on admission was 9.53 ± 2.06, and the preoperative hematoma volume was 40.58 ml ± 7.07 ml. **Craniotomy group**: the age of the patients was 59.58 ± 12.51 years, with 34 (68.00%) male patients and 16 (32.00%) female patients. There were 3 patients (6.00%) with coronary artery disease, 27 patients (54.00%) with hypertension and 9 patients (18.00%) with diabetes mellitus. The GCS on admission was 9.58 ± 1.92, and the preoperative hematoma volume was 42.77 ± 9.13 ml. **Endoscopic group**: the age of the patients was 62.85 ± 11.36 years, with 12 male patients (60.00%) and 8 female patients (40.00%). There was 1 patient with coronary artery disease (5.00%), 11 patients with hypertension (55.00%) and 5 patients with diabetes mellitus (25.00%). The GCS on admission was 9.55 ± 1.76 and the preoperative hematoma volume was 41.30 ± 11.88 ml. There were no statistically significant difference in the demographic as well as clinical data between the ROSA group and the craniotomy group and the endoscopy group (Tables 1 and 2).

**Table 1:** Baseline characteristics and treatment outcomes were compared between the ROSA group and the craniotomy group

Variables	Total (n = 90)	Craniotomy group(n = 50)	ROSA group (n = 40)	*P*
Age(years), Mean ± SD	59.68 ± 12.45	59.58 ± 12.51	59.80 ± 12.53	0.652
Sex, n(%)				0.764
Male	60 (66.67)	34 (68.00)	26 (65.00)	
Female	30 (33.33)	16 (32.00)	14 (35.00)	
GCS at admission, Mean ± SD	9.56 ± 1.97	9.58 ± 1.92	9.53 ± 2.06	0.901
Hematoma volume(ml), Mean ± SD	41.79 ± 8.31	42.77 ± 9.13	40.58 ± 7.07	0.564
Coronary heart disease, n(%)				1.000
No	84 (93.33)	47 (94.00)	37 (92.50)	
Yes	6 (6.67)	3 (6.00)	3 (7.50)	
Diabetes, n(%)				0.474
No	76 (84.44)	41 (82.00)	35 (87.50)	
Yes	14 (15.56)	9 (18.00)	5 (12.50)	
Hypertension, n(%)				0.292
No	37 (41.11)	23 (46.00)	14 (35.00)	
Yes	53 (58.89)	27 (54.00)	26 (65.00)	
Interval between stroke and surgery(h) Mean ± SD	49.24 ± 19.41	47.64 ± 11.55	51.25 ± 11.05	0.137
Operation time(min), Mean ± SD	170.58 ± 55.69	184.88 ± 63.13	152.70 ± 38.46	**0.007**
Intraoperative blood loss > 50 (ml), n(%)				** < 0.001**
No	37 (41.11)	4 (8.00)	33 (82.50)	
Yes	53 (58.89)	46 (92.00)	7 (17.50)	
Intraoperative blood transfusion, n(%)				0.504
No	85 (94.44)	46 (92.00)	39 (97.50)	
Yes	5 (5.56)	4 (8.00)	1 (2.50)	
Hematoma clearance rate(%), Mean ± SD	90.91 ± 4.01	89.41 ± 3.47	92.78 ± 3.87	** < 0.001**
Postoperative pulmonary infection, n(%)				**0.007**
No	67 (74.44)	31 (62.00)	35 (87.50)	
Yes	23 (25.56)	19 (38.00)	5 (12.50)	
Postoperative urinary tract infection, n(%)				0.504
No	85 (94.44)	46 (92.00)	39 (97.50)	
Yes	5 (5.56)	4 (8.00)	1 (2.50)	
Postoperative Intracranial infection, n(%)				0.077
No	81 (90.00)	42 (84.00)	39 (97.50)	
Yes	9 (10.00)	8 (16.00)	1 (2.50)	
Postoperative rebleeding, n(%)				0.501
No	88 (97.78)	48 (96.00)	40 (100.00)	
Yes	2 (2.22)	2 (4.00)	0 (0.00)	
Postoperative ventilator-assisted ventilation > 96H, n(%)				0.897
No	76 (84.44)	42 (84.00)	34 (85.00)	
Yes	14 (15.56)	8 (16.00)	6 (15.00)	
Postoperative death, n(%)				1.000
No	87 (96.67)	48 (96.00)	39 (97.50)	
Yes	3 (3.33)	2 (4.00)	1 (2.50)	
Surgical fee(w), Mean ± SD	1.45 ± 1.13	1.38 ± 1.44	1.53 ± 0.54	** < 0.001**
Time from surgery to discharge(d), Mean ± SD	24.30 ± 16.37	25.08 ± 16.54	23.32 ± 16.31	0.477
Hospitalization fee(w), Mean ± SD	12.88 ± 9.33	13.96 ± 10.46	11.53 ± 7.59	0.214
mRS scores at discharge, Mean ± SD	2.83 ± 1.17	3.08 ± 1.24	2.53 ± 1.01	**0.038**
> 3 months after discharge mRS, Mean ± SD	2.76 ± 1.13	3.00 ± 1.25	2.45 ± 0.90	**0.018**

**Table 2:** Baseline characteristics and treatment outcomes were compared between the ROSA group and the Endoscopic group

Variables	Total (n = 60)	Endoscopic group (n = 20)	ROSA group (n = 40)	*P*
Age(years), Mean ± SD	60.82 ± 12.14	62.85 ± 11.36	59.80 ± 12.53	0.428
Sex, n(%)				0.705
Male	38 (63.33)	12 (60.00)	26 (65.00)	
Female	22 (36.67)	8 (40.00)	14 (35.00)	
GCS at admission, Mean ± SD	9.53 ± 1.95	9.55 ± 1.76	9.53 ± 2.06	0.835
Hematoma volume(ml), Mean ± SD	40.82 ± 8.87	41.30 ± 11.88	40.58 ± 7.07	0.405
Coronary heart disease, n(%)				1.000
No	56 (93.33)	19 (95.00)	37 (92.50)	
Yes	4 (6.67)	1 (5.00)	3 (7.50)	
Diabetes, n(%)				0.391
No	50 (83.33)	15 (75.00)	35 (87.50)	
Yes	10 (16.67)	5 (25.00)	5 (12.50)	
Hypertension, n(%)				0.453
No	23 (38.33)	9 (45.00)	14 (35.00)	
Yes	37 (61.67)	11 (55.00)	26 (65.00)	
Interval between stroke and surgery(h) Mean ± SD	51.55 ± 12.24	52.15 ± 14.63	51.25 ± 11.05	0.791
Operation time(min), Mean ± SD	175.50 ± 56.57	221.10 ± 60.14	152.70 ± 38.46	** < .001**
Intraoperative blood loss > 50 ml, n(%)				** < .001**
No	38 (63.33)	5 (25.00)	33 (82.50)	
Yes	22 (36.67)	15 (75.00)	7 (17.50)	
Intraoperative blood transfusion, n(%)				** < .001**
No	51 (85.00)	12 (60.00)	39 (97.50)	
Yes	9 (15.00)	8 (40.00)	1 (2.50)	
Hematoma clearance rate(%), Mean ± SD	92.10 ± 4.01	90.76 ± 4.02	92.76 ± 3.88	**0.038**
Postoperative pulmonary infection, n(%)				0.194
No	49 (81.67)	14 (70.00)	35 (87.50)	
Yes	13 (21.67)	6 (30.00)	5 (12.50)	
Postoperative urinary tract infection, n(%)				0.530
No	57 (95.00)	18 (90.00)	39 (97.50)	
Yes	3 (5.00)	2 (10.00)	1 (2.50)	
Postoperative Intracranial infection, n(%)				1.000
No	58 (96.67)	19 (95.00)	39 (97.50)	
Yes	2 (3.33)	1 (5.00)	1 (2.50)	
Postoperative rebleeding, n(%)				
No	0(0%)	0(0%)	0(0%)	
Yes	0(0%)	0(0%)	0(0%)	
Postoperative ventilator-assisted ventilation > 96H, n(%)				0.555
No	49 (81.67)	15 (75.00)	34 (85.00)	
Yes	11 (18.33)	5 (25.00)	6 (15.00)	
Postoperative death, n(%)				0.069
No	55 (91.67)	16 (80.00)	39 (97.50)	
Yes	5 (8.33)	4 (20.00)	1 (2.50)	
Surgical fee(w), Mean ± SD	1.56 ± 0.91	1.61 ± 1.41	1.53 ± 0.54	0.089
Time from surgery to discharge(d), Mean ± SD	26.33 ± 21.67	32.35 ± 29.24	23.33 ± 16.31	0.649
Hospitalization fee(w), Mean ± SD	12.19 ± 7.13	13.52 ± 6.05	11.53 ± 7.59	0.086
mRS scores at discharge, Mean ± SD	2.70 ± 1.25	3.05 ± 1.61	2.53 ± 1.01	0.396
> 3 months after discharge mRS, Mean ± SD	2.57 ± 1.23	2.80 ± 1.70	2.45 ± 0.90	0.877

### Comparison of Surgical Data as Well as Prognosis Between the ROSA Group and the Craniotomy Group (Table 1)

#### Surgical Data

Compared with the craniotomy group, the ROSA group had a shorter operation time (152.70 ± 38.46 min vs. 184.88 ± 63.13 min), lower intraoperative blood loss > 50 ml (17.50% vs. 92.00%), higher rate of hematoma clearance (92.78% ± 3.87% vs. 89.41% ± 3.47%), and lower rate of intraoperative blood transfusion (2.5% vs. 8%). The operation time (*p* = 0.007), intraoperative blood loss > 50 mL (*p* < 0.001), and hematoma clearance rate (*p* < 0.001) (Table 1) showed statistically significant difference among the groups.

#### Postoperative Data

Compared with the craniotomy group, the ROSA group showed lower rates of postoperative pulmonary infection (12.5% vs. 38%), postoperative intracranial infection (2.5% vs. 16%), postoperative urinary tract infection (2.5% vs. 8%), postoperative rebleeding (0% vs. 4%), and postoperative death (2.5% vs. 4%). The rate of postoperative ventilator-assisted ventilation > 96 h was higher in the ROSA group than in the craniotomy group. The mRS scores at discharge (2.53 ± 1.01 vs. 3.08 ± 1.24) and > 3 months after discharge (2.45 ± 0.90 vs. 3.00 ± 1.25) were lower in the ROSA group than those in the craniotomy group. The rates of postoperative lung infection (*p* = 0.007) and mRS scores at discharge (*p* = 0.038) and > 3 months after discharge (*p* = 0.018) showed statistically significant difference among the groups. (Table 1).

#### Other Data

The surgical fee was significantly higher in the ROSA group than in the craniotomy group (1.53 ± 0.54 w vs. 1.38 ± 1.44 w, *p* < 0.001). However, the craniotomy group had a higher hospitalization fee (11.53 w ± 7.59 w < 13.96 w ± 10.46 w) and longer time from surgery to discharge (23.32 d ± 16.31d vs. 25.08 d ± 16.54 d) (Table 1).

### Comparison of Surgical Data as Well as Prognosis Between the ROSA Group and the Endoscopic Group (Table 2)

#### Surgical Data

Compared with the endoscopy group, the ROSA group had a shorter operation time (152.70 min ± 38.46 min vs. 221.10 min ± 60.14 min), and lower rates of intraoperative blood loss > 50 mL (17.50% vs. 75.00%) and intraoperative blood transfusion (2.5% vs. 40%). The operation time (*p* < 0.001), intraoperative blood loss > 50 mL (*p* < 0.001), and intraoperative blood transfusion rate (*p* < 0.001) showed statistically significant difference among the groups.

#### Postoperative Data

Compared with the endoscopy group, the ROSA group showed a higher hematoma clearance rate (92.76% ± 3.88% vs. 90.76% ± 4.02%). Similarly, compared with the endoscopy group, the ROSA group showed lower rates of postoperative pulmonary infection (12.5% vs. 30%), postoperative intracranial sensation (2.5% vs. 5%), postoperative urinary tract infection (2.5% vs. 10%), postoperative death (2.5% vs. 20%), and postoperative ventilator-assisted ventilation > 96 h. The mRS scores on discharge (2.53 ± 1.01 vs. 3.05 ± 1.61) and > 3 months after discharge (2.45 ± 0.90 vs. 2.80 ± 1.70) were lower than the endoscopy group. The hematoma clearance rates showed statistically significant difference among the groups. (*p* = 0.038).

#### Other Data

Compared with the endoscopy group, the ROSA group showed lower surgical (1.53w ± 0.54w > 1.61w ± 1.41w) and hospitalization (11.53 w ± 7.59 w < 13.52 w ± 6.05 w) fees, as well as a shorter time from surgery to discharge (23.33 ± 16.31 d vs. 32.35 ± 29.24 d). However, these differences were not statistically significant.

## Discussion

Between 1990 and 2010, the global incidence of cerebral hemorrhage increased by 47%, with the highest incidence concentrated in low-income countries [1]. Hematoma following a cerebral hemorrhage exerts occupying effects, increasing intracranial pressure and causing midline shift, which can damage surrounding brain tissues and even affect critical areas such as the brainstem. Additionally, the leaked blood can release neurotoxins, and proinflammatory factors may contribute to secondary brain injury [9, 10].

Craniotomy can directly remove cerebral hematoma, reduce the occupying effect of cerebral hemorrhage and its surrounding cerebral edema, and decrease damage to brain tissue caused by degradation products in the hematoma. Surgeon can quickly and effectively control bleeding during the procedure, reducing the risk of rebleeding. However, craniotomy is typically a high-trauma surgery with prolonged operation times, significant intraoperative bleeding, and a rebound in intracranial pressure 24 to 48 h post-surgery. Additionally, challenges in wound healing can lead to postoperative infections and complications, including hydrocephalus [11, 12]. Additionally, some patients require a second surgery for cranial repair, which increases pain and financial burden [13]. Minimally invasive hematoma removal has been widely performed in recent years. Neuroendoscopic hematoma removal requires only a small bone window in the skull to remove the hematoma, and the endoscope provides a high-definition field of view, limiting patient trauma and effectively reducing intraoperative damage to the surrounding brain tissue, blood vessels, and nerves [14]. However, neuroendoscopy relies on CT markers for surface localization, which involves hand-drawn positioning and may result in poor puncture accuracy. The endoscopic view is two-dimensional, lacking depth perception, which can lead to potential image distortion. Additionally, the limited space for endoscopic surgery makes it challenging, as the endoscope is prone to blood contamination and requires frequent adjustment of the lens angle. As a result, endoscopic hematoma removal demands a high level of surgical skill and precision.

The ROSA is a multifunctional surgical robot developed by the French company MedTech. This robot has gradually been used in recent years for deep intracranial electrode implantation and intracerebral tumor localization and biopsy, and is known as the “Leonardo da Vinci” surgical robot in the neurosurgical field [15]. Our experiences with the ROSA robot have revealed the following unique advantages in removing cerebral hematomas compared with craniotomy and neuroendoscopy. 1. The ROSA robot can accurately locate and display the morphology of cerebral hematomas. In the preoperative localization of cerebral hematomas, the ROSA robot can integrate preoperative craniocerebral CT, computed tomography angiography (CTA), magnetic resonance angiography (MRA), and other high-quality images to show three-dimensional images of the morphology of the hematoma and its relationship with the surrounding blood vessels. 2. When removing cerebral hematomas, the ROSA robot has a small operating error and a high level of surgical safety. Additionally, it features four registration and alignment modes (body surface marking point registration, cranial implantation marking point registration, frame marking point registration, and automatic registration of the laser without marking points); thus, surgeons can select the appropriate registration mode according to surgery requirements. The operating error of ROSA is < 1 mm, with a minimum error in cranial marking during deep brain electrical stimulation of 0.42 mm [16]. When removing a cerebral hematoma, surgeons can combine the three-dimensional images of the hematoma and the surrounding blood vessels to develop a surgical plan and select surgical parameters (e.g., target point and surgical access). ROSA can also automatically and accurately locate the long axis of the hematoma according to the shape and volume of the hematoma reconstructed by the three-dimensional reconstruction. Thus, the different registration modes allow surgeons to perform high-precision procedures, which can minimize the risk of damage to the surrounding brain tissue and blood vessels [17]. 3. The ROSA robot has fewer surgical steps, better surgical effects, and shorter surgical time; moreover, it does not require installing additional instruments before surgery. The ROSA robotic arm has a wide range of intraoperative operations; with 360° six-dimensional degrees of freedom and automatic sensing, repeated adjustment of the angle is not required and the coordinates of the surgical access are not constrained. Thus, theoretically, no surgical blind spot or dead angle was observed. After the surgeon sets the target point and surgical path, the robotic arm automatically performs hematoma localization and puncture [18]. After most of the hematoma is removed, the surgeon can use warm saline to repeatedly wash away the residual hematoma to ensure its complete removal. It is important to note that during hematoma evacuation, the surgical cavity may collapse, and the hematoma's location or surrounding brain tissue structure may shift. However, the ROSA robot currently lacks the ability to navigate the entire process and provide real-time feedback on these changes. Additionally, unlike endoscopy, the ROSA robot does not offer direct visualization of the surgical field, making it challenging to address recurrent bleeding promptly during surgery. To mitigate these limitations, we typically plan a more detailed surgical approach in advance, including multi-point localization of the hematoma, considering both deep and superficial locations, while avoiding cerebral sulci and ventricles. If the hematoma shifts during the procedure, we use an ultrasound probe to re-localize its position and adjust the surgical approach as needed to remove the hematoma effectively.

Longer operative durations in neurosurgical procedures expose open wounds to the external environment for a longer period, thus increasing the risk of surgical site infections [19, 20]. Qin et al. reported that increased surgical times independently increased the risk of postoperative tract infections [21]. Prolonged operating times also increase surgeon fatigue and surgical error rates. The ROSA has fewer steps, is less invasive, and eliminates the need for multiple instrument adjustments, which effectively reduces the operating time and the risk of postoperative infections. Postoperative infection is one of the most life-threatening infections in patients undergoing neurosurgery and a key determinant of their short- and long-term prognoses [22]. Regardless of the severity, infection prolongs surgical incision healing, may exacerbate the patient's condition, and increases the patient's length of hospital stay, hospitalization expenses, and readmission rate [23, 24]. In the present study, the rates of postoperative pulmonary, urinary tract, and intracranial infections were lower in the ROSA group than in the craniotomy and endoscopy groups. Additionally, the rate of postoperative pneumonia was significantly lower in the ROSA group than those in the craniotomy and endoscopy groups (12.5% vs. 38% vs. 30%). This finding was related to the short operation time in the ROSA group, mainly owing to the reduced tracheal intubation time, decreased intraoperative bleeding so that poor nutritional status during the postoperative period was avoided, and lower intraoperative trauma so that the patients could heal more rapidly. Among the three groups, the ROSA group had the lowest postoperative mortality, as well as lower mRS scores at discharge and > 3 months after discharge. We attributed this difference to the frameless design of the ROSA device. Moreover, self-localization of the hematoma and puncture by the robotic arm during the operation allowed more operator flexibility, and ensured its ease of use. Three-dimensional reconstruction of the hematoma allowed the surgeons to better remove the hematoma and avoid damaging other blood vessels and surrounding brain tissue, thus reducing the occurrence of postoperative complications and improving patient prognosis.

Our study has several limitations: (1) it is a retrospective analysis based on data from a single center, which may introduce potential bias; (2) the use of the ROSA robot for hematoma removal is a relatively recent development in neurosurgery, resulting in a limited sample size from a single source, which restricts the scope of statistical analysis. Larger, multi-center studies are needed to validate these findings.

## Conclusion

Compared with craniotomy and neuroendoscopic hematoma removal, the ROSA robot allowed more precise, rapid, and safe cerebral hematoma removal. This technique also effectively reduced the occurrence of postoperative complications and improved patient prognosis. Thus, the ROSA robot has great application prospects and warrants further clinical application.

## Supplementary Information

Below is the link to the electronic supplementary material.

Supplementary file1 (XLSX 26 KB)

## Data Availability

All data generated or analysed during this study are included in this published article (and its Supplementary Information files).
